# Evaluating the effectiveness and acceptability of two positive body image media micro-interventions among children aged 4–6 years old – a study protocol

**DOI:** 10.1186/s12889-024-20869-z

**Published:** 2024-12-19

**Authors:** H. G. Smith, K. M. Garbett, P. White, H. Williamson, N. Craddock

**Affiliations:** School of Social Sciences, Centre for Appearance Research, University of the West of England, Coldharbour Lane, Bristol, BS16 1QY UK

**Keywords:** Children, Positive body image, Micro-interventions, Weight bias, Prevention, Body appreciation, Functionality appreciation, Children’s media

## Abstract

**Background:**

Children’s online media perpetuates appearance idealised images and can negatively impact the way children feel about their own and other people’s bodies (e.g., weight bias) at a young age. The development and evaluation of body image interventions for young children to counteract this, are scarce. There is a need for prevention efforts to nurture the development of positive body image among this group to help mitigate potential body image concerns in later childhood. Media-based approaches promoting positive body image messages have shown preliminary efficacy. In collaboration with industry partners, we have developed two positive body image media micro-interventions (a 15-minute episode and a music video) to be evaluated in a fully powered RCT.

**Methods:**

We aim to recruit 440 children between the ages of 4 and 6 years to be randomised into one of four conditions: (i) 15-minute episode intervention, (ii) 15-minute episode control, (iii) 3-minute music video intervention, or (iv) 3-minute music video control. This study will be conducted face-to-face, whereby children and a parent attend a media screening session and children complete pre-and post-intervention measures of positive body image and weight bias. Both the child and parent will watch their assigned media, together on a tablet device. Due to their age, children will complete outcome measures with a trained moderator in a play-based interview pre-intervention (T1), immediately post-intervention (T2) and one-week follow up (T3). A corresponding parent will complete a questionnaire on intervention acceptability at T2, and re-watch of their assigned media at T3. The primary outcome will be the change in body appreciation, and secondary outcomes include change in functionality appreciation and weight bias. Exploratory analyses will determine any effect of gender (girls vs. boys), year group (reception vs. year 1) dosage or delayed effects. Moderator fidelity will be also assessed.

**Discussion:**

This study will evaluate two positive body image micro-interventions among children 4–6 years old. These interventions have the potential to bolster children’s positive body image and reduce weight bias. A dissemination plan is in place with project stakeholders such that the interventions can reach millions of children worldwide.

**Trial registration:**

The trial is registered with Clinical Trial.gov, Ref number: NCT06146647.

**Supplementary Information:**

The online version contains supplementary material available at 10.1186/s12889-024-20869-z.

## Background

### Body image in childhood

Body image – the way people think, feel, and behave in relation to their body – encompasses two distinct constructs; negative body image (i.e., body dissatisfaction) and positive body image (i.e., an overarching love, respect, appreciation for, and acceptance of the body and what it can do [1]). Traditionally, body dissatisfaction has been viewed as a problem that develops during adolescence; however, such concerns may develop much earlier than this [2, 3]. Typically, very young children (e.g., ages 3–5 years) express positive feelings towards their bodies [4], with some beginning to report body dissatisfaction during early years of schooling [5]. Negative attitudes towards other people’s bodies, particularly weight bias, can emerge as early as three years ( [6]). By age five years, many children have internalised appearance ideals [7] and by age six, a large proportion of children (up to 70%) report dissatisfaction with specific body parts, weight and/or shape [8, 9], and a desire to be thinner or more muscular for girls and boys respectively [5, 10]. Body dissatisfaction becomes problematic when high value or investment is placed upon appearance [11]. Appearance investment often peaks as children emerge into pre-adolescence, a period when body dissatisfaction can become entrenched [12]. Together, this highlights a small but unique window of opportunity to shape and nurture the development of positive body image among younger children to harness their existing positive attitudes and potentially prevent the future onset of body dissatisfaction.

### The role and impact of the media

Sociocultural theory (Tripartite Model of Influence^1^; [13]) suggests children’s media, particularly those promoting appearance-idealised messaging, significantly influences the onset and development of body dissatisfaction. Children’s media plays a pivotal role in learning [14] including teaching children about beauty and the value of appearance. Content analyses of children’s TV, music, and film over the past few decades underscores the prevalence of characters who conform to cultural appearance ideals with female characters being thin or slender (e.g., *Disney princesses like Elsa from Frozen)* and male characters being muscular, strong, and athletic, such as characters like *the Incredibles or* superheroes *like Spiderman* [15–17]. Positive qualities such as being attractive (and having love interests), popular, happy, and intelligent are often attributed to thinner characters [18, 19]. In contrast, larger characters are less frequently seen on screen and more likely to be portrayed negatively [20]; have limited social connections, be subject to teasing, considered lazy, depicted as less intellectually capable, or characterised as a villain [21–23]. A few examples include, *Augustus Gloop* from *Charlie and the Chocolate Factory*, *Ursula the Sea Witch*,* and The Hunchback of Notre Dame*. This is also evidenced by data with a recent study highlighting 84% of children’s top grossing films from 2012 to 2015 included verbal insults about body size or weight [24]. As the Tripartite Model [13] suggests, these messages become internalised, and repeat exposure to this type of media reinforces gendered appearance stereotypes [25].

In recent years, where young children go to consume media content has proliferated. In addition to TV viewing, children now have unlimited access to media through online platforms such as Netflix and YouTube (i.e., YouTube Kids). A recent survey found 80% of children aged 0–7 years were subscribed to YouTube, spending an average 1.4 h per day consuming media content [26]. YouTube not only offers long form content in the style of TV episodes but also a plethora of short form content (e.g., music videos) which has become the most popular form of children’s content on YouTube [27]. Notably, ‘*Baby Shark’*, a 2-minute 17-second music video for children, became the first YouTube video to reach over 10 billion views [28] and the most watched video on the platform.

Since the COVID-19 pandemic, streaming platforms such as YouTube, have also become an online learning tool for children, offering interactive videos from popular children’s shows on varied topics including mathematics, languages, understanding emotions, and friendships [29]. However, a large proportion of this content, including animation and cartoons, features appearance-idealistic themes such as positive messaging about being thin and attractive [20, 30]. Others use humour to promote prejudiced attitudes, much like media elsewhere. For example, ‘*Peppa Pig’*, an animated show which follows Peppa’s adventures with her family and friends and teaches children about daily activities, includes characters who engage in negative body talk, exhibiting weight biased attitudes. For example, Peppa tells Daddy pig he needs to get fit as he is ‘a bit too fat’, making jokes about his ‘big tummy’; when Daddy Pig tries to join Peppa in her treehouse he gets wedged in the door whilst the other characters laugh (episode 37 or 42, season 1).

Frequent exposure to media promoting appearance-idealised images and weight stigmatising messages comes with consequences for children and adolescents, especially children who are higher weight. Higher weight children are stigmatised in school and recreational settings (e.g., have less friends, be the victims of teasing and bullying [31]), which can lead to increased social isolation, more internalised weight bias, and the exacerbation of body dissatisfaction [32, 33]. More generally, body dissatisfaction can lead to other psychological and social concerns as children transition from childhood into adolescence including elevated stress, anxiety, low mood, disordered eating (e.g., dietary restraint; [34]), decreased academic achievement and increased disengagement from various aspects of life (e.g., dropping out of sport and avoiding social interactions; [35]).

### Strategies and interventions for children

Despite Smolak and Levine’s ( [36]) call for prevention efforts to promote positive body image among young children more than two decades ago, progress has been limited. Targeting body dissatisfaction is challenging once concerns are entrenched; intervention strategies tend to be lengthy, costly, and intensive, only a handful have proven efficacy and there are additional barriers (e.g., funding, access) to implementation [37]. Instead, experts recommend shifting the focus to prevention at a group level among younger demographics, so that a whole population is less exposed to and more resilient to risk factors [38]. One way to achieve this is to bolster children’s positive body image by broadening their conceptualisation, and acceptance of diverse appearances [35] and foster resilience to appearance-ideal messages ( [3, 39]). Consequently, this serves as a protective factor against the adverse effects of appearance-idealised media exposure.

Leveraging media is a key strategy for young children, as storytelling and role modelling are primary avenues for learning [40]. Numerous TV shows have successfully adopted this approach to enhance children’s empathy, self-efficacy, emotional competence, school readiness and pro-social behaviour; *Sesame St* [41], *Daniels Tiger in the Neighbourhood* [42], and *Blues Clues* [43]. Moreover, there has been a recent surge in media content, particularly within the Disney franchise, aiming to increase representation more broadly. This is exemplified by the release of films like *Moana*, *Encanto*, and *Coco* which feature characters, particularly protagonists, from diverse ethnic backgrounds and a range of body sizes [44]. However, such content has not been empirically evaluated.

To date, three media-based interventions designed to foster positive body image among young children (i.e., 9 years and under) have been empirically evaluated. The first study evaluated ‘*Shapesville’*, a picture based book promoting self-acceptance and appearance diversity, among girls aged 5–9 years [45]. *Shapesville* was found to generate immediate improvements in appearance satisfaction which were maintained at 6-weeks follow up, relative to a waitlist control. Most interestingly, girls’ recognition of their own special talents, a positive body image theme woven into the book's storyline, significantly increased at post-intervention relative to the control group. The impact of the book on young boys’ body image and related attitudes is unknown. The second study evaluated a series of 60-second animated ‘micro-interventions’ centred around the children’s TV series *‘Steven Universe and the Crystal Gems’* [46]. The videos targeted risk and protective factors for body image among boys and girls aged 7–10 years and resulted in immediate improvements in state body satisfaction and sustained effects in trait media literacy and appearance teasing. Unexpectedly, the control performed in line with the intervention condition. This finding was attributed to the inclusion of characters of diverse body shapes and sizes in the control episode, indicating that representation of diverse body sizes on its own could be a successful strategy to improve children’s body image. The third study evaluated a theatrical production of ‘*Cinderella – The AWESOME Truth!’* [47], designed to increase children’s (aged 5–9 years) body appreciation. Despite the lack of a control group in the study’s research design, the performance was associated with improved state body appreciation, self-perceived uniqueness and self-perceived ‘awesomeness’.

#### Micro-interventions

Together these studies highlight the potential of embedding positive body image messaging within different forms of children’s media to foster positive body image and in-particular the innovative use of micro-interventions. Micro-interventions are low-intensity, brief interventions, designed to deliver in the moment (e.g., state-based) benefits to an individual’s mental health [46, 48]. Micro-interventions are particularly well suited for children due to their limited attention span and developmental capacity ( [46]) and have proven efficacy in delivering immediate benefits to pre-adolescent’s and adolescent’s body image (e.g [49, 50]). They can also be easily integrated into media environments that children are avid consumers of, such as YouTube, however the use of micro-interventions among young children is yet to be explored.

### Aims

The aims of this paper are to (1) outline the development of two positive body image media micro-interventions (a 15-minute episode and 3-minute music video) designed for children aged 4–6-year-olds, (2) describe how these interventions will be evaluated, (3) detail the plans for dissemination, and (4) discuss the study’s potential strengths and limitations.

The described study is a randomised controlled trial which aims to assess the effectiveness and acceptability of the two interventions among young children. Our primary research questions and hypotheses are:

- RQ1. Relative to time-matched active controls, are the two positive body image media micro-interventions effective in yielding immediate improvements in children’s body appreciation (primary outcome) and functionality appreciation and in reducing weight bias (secondary outcomes)?
- H1: We anticipate children randomised into one of the intervention conditions to experience an immediate improvement (T2) in body appreciation and functionality appreciation, relative to the control groups. We also expect the intervention groups to report an immediate reduction in weight bias (T2), relative to the control groups.
- RQ2. Are the two positive body image media micro-interventions acceptable to children and their participating parent/guardian?
- H2. We expect the two positive body image media micro-interventions to be enjoyed by children and viewed as age appropriate and relevant by their participating parent/guardian.

In addition, to our knowledge, these are the first media-based interventions targeting positive body image, designed for very young children. As such, we plan to also test five secondary exploratory research questions, examining two hypothesised moderators, the effects of exposure to positive body image media content via repeat watching and due to the duration of the media, as well as sustained effects.

#### Moderators

- RQ3. Does gender moderate intervention effectiveness?
- H3. We predict that the interventions will be more effective for girls than for boys.
- RQ4. Does year group moderate intervention effectiveness?
- H4. We predict that the interventions will be more effective for children in Year 1 (ages 5 and 6) than children in Reception (ages 4 and 5).

#### Exposure effects

- RQ5. Are there within-group intervention effects whereby repeated exposure to either one of the positive body image media micro-interventions is positively correlated with improvements at T3?
- H5. We expect that children within each intervention condition (e.g., 15-minute episode and music video) will experience greater improvements to functionality appreciation and body appreciation and greater reductions in weight bias, at one-week follow up (T3) the more they have watched their content from T2 to T3.
- RQ6. Is there a difference in terms of the immediate pre-post effectiveness outcomes between the two interventions?
- H6. We anticipate the 15-minute intervention will be more effective than the music video on the premise that there will be greater exposure to core messaging.

#### Sustained effects

- RQ7. Do we observe sustained effects approximately one week following media exposure, controlling for repeat watching?
- H7. We anticipate children to report sustained effects at one-week follow up (T3), relative to the control groups based on parent-child conversations on topics related to positive body image.

## Method

### Study design

**Fig. 1:**
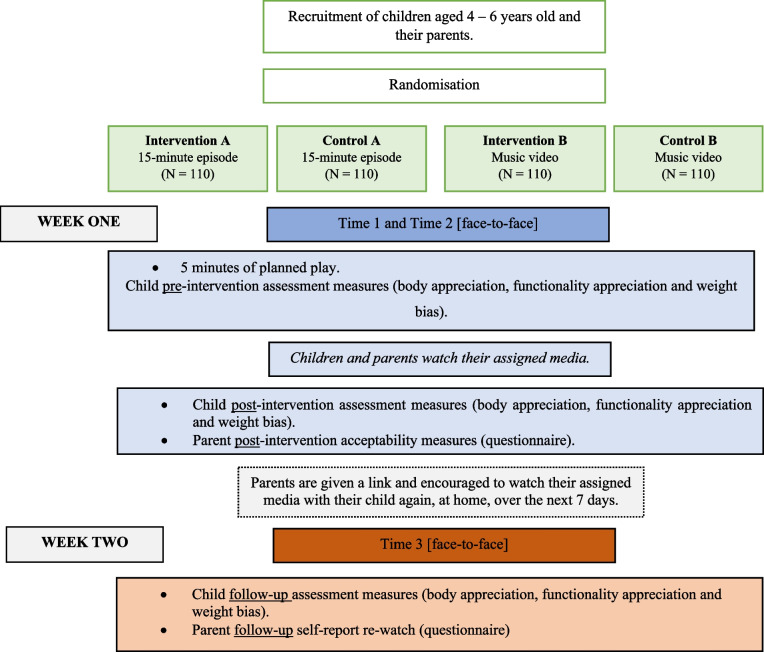
Study design flowchart

A 4-armed randomised controlled trial (RCT) will be conducted to evaluate two media micro-interventions: an approximately 15-minute positive body image episode and a 3-minute positive body image music video. Participants will be randomised using block randomisation, stratified by gender and year group into one of four conditions: (i) 15-minute episode intervention, (ii) 15-minute episode control, (iii) 3-minute music video intervention, or (iv) 3-minute music video control. Randomisation blocks will be in groups of four. The primary outcome for intervention effectiveness is body appreciation, assessed among children pre- and immediately post-intervention and at one-week follow up (T1, T2 and T3). The secondary outcomes for intervention effectiveness are functionality appreciation and weight bias, which will also be assessed among children at T1, T2 and T3. Parents and children will report on intervention acceptability at T2, and parents will report on media re-watch at T3. Parents will provide demographic information for them and their child (T0). A diagram of the research process is presented in Fig. 1.

#### Pilot study

A small internal pilot study was conducted at the end of 2023 for the purpose of trialling the research design and protocols. A stop-go protocol was implemented, and progression criteria was based on; (1) feasibility assessment such as participant recruitment, retention, and evidence of ceiling/floor effects on the primary outcome measure and (2) harm assessment including evidence of child distress due to the content / research process reported by children, parents, or moderators. Criteria was rated for each parameter with a traffic light system of green, amber, and red as seen in Table 1.

**Table 1:** Stop-go progression criteria

Criteria			Green	Amber	Red
1. ***Feasibility assessment***					
Participant recruitment	*How many participants showed up at T1?*		**More than 80%** Continue to main trial	**Between 60–80%** Consult research team and review recruitment plans	**Less than 60%** Pilot study may need to be extended
Participant retention at T3	*How many participants showed up at T3?*		**More than 80%** Continue to main trial	**Between 50–80%** Consult research team and discuss strategies to increase retention	**Less than 50%** The main trial will need to reconsider the inclusion of T3
Ceiling and/or floor effects on the primary outcome	*Is there evidence of ceiling or floor effects at T1 on the primary outcome measure*,* child body appreciation?*		**No** Continue to main trial	**Partial** Consult research team and discuss potential measure amendments	**Yes** The main trial will need to potentially reconsider the response scale for the primary outcome
2. ***Harm assessment***					
Child distress	*Were any of the children visibility distressed?*,* did any of the children tell the moderators anything concerning?*,* did any parent withdraw their child during the pilot due to the content or research process?*		**None** Continue to main trial	**Between 1–10%** Review feedback from moderators and parents, consult with the research team	**More than 10%** All cases to be qualitatively investigated and the team to consider the safety of the trial

A total of 80 children were enrolled, of which 63 attended. On the day, one child refused to take part, and one child struggled to focus on the tasks making it hard for them to participate, resulting in usable data from 61 children and their accompanying parent. Insights from the pilot allowed us to modify the moderator guide to specify what needed to be said verbatim, versus what could be adapted to suit the individual needs of the child. Additionally, the option for children to choose which order to complete each game/measure was removed to streamline the procedure and reduce participant time burden. Further, we identified ways to reduce the influence of the moderator on children’s responses, by randomising the order of the weight bias images, instructing moderators to not pass comment during the weight bias activity (e.g., “thank you”, “great”), and throughout the moderation, avoiding the use of ticks, crosses, or red pen on their moderator forms.

### Intervention description and development

The content for the media micro-interventions is rooted in principles of Positive Body Image Theory ( [1, 51]), encompassing three main themes. First, functionality appreciation aims to teach children to recognise and appreciate the things their body can do regardless of its appearance ( [52]). The second theme is mindful attunement, highlighting the connection between internal and external experiences and tuning in to the body’s signals ( [53]). Third, acceptance of diverse appearances encourages children to understand and celebrate that people come in different body shapes and sizes and everyone is of equal worth ( [38]). Specifically, the content of these interventions aims to reduce weight bias by disrupting children’s beliefs about body shape and size. These themes are achieved explicitly through the episode's script and music lyrics and implicitly via diverse casting and a wide representation of characters on screen.

The micro-interventions described below were developed through a multi-stakeholder collaboration with the Dove Self-Esteem Project (the social purpose initiative of Unilever’s largest personal care brand, Dove), children’s entertainment company and creators of Blippi, Moonbug Entertainment, and body image researchers from the Centre for Appearance Research, University of the West of England, Bristol. Blippi is a global children’s programme with over 22 million subscribers on YouTube (as of December 2024). Blippi provides educational learning for children aged 4 years old and upwards via fun and interactive videos.

#### Micro-intervention 1: ‘Blippi’s Wonderful Talent Show’ – digital series 

‘Blippi’s Wonderful Talent Show’ is a series of four short episodes (approximately 15 min each), hosted on YouTube. Each episode is designed to teach children about the wonderful things their bodies can do, recognise that bodies come in different shapes and sizes, and celebrate what is unique about themselves and others. The series features fun-loving Blippi and his co-star Meekah alongside a cast of children and ‘experts’ who discover their own special talents in the build- up to a talent show finale. The episodes are intended to be co-watched with a parent and are interactive (e.g., Meekah speaks to the audience and says *‘I like to move my arms and legs like this when I’m happy! Hey*,* which body part do YOU like to dance with best*? *[pause for response]*’ and Blippi addresses the audience by saying *‘my body is SO silly awesome! (to viewers)*,* Hey*,* will you try with me? ).*

This study will evaluate the effectiveness and acceptability of the first episode in the series,

‘Dance Your Own Way’ which features Blippi and Meekah alongside two dancing protagonists; a dancing expert, Angel (a male in a larger body), a child dancer, Lono (a boy in a larger body, aged 11). Lono discovers what his body can do through dance and learns how to master the pirouette with the help of Angel and friends. There are also two additional child characters; one girl aged 10 and a boy aged 11. The central aim is for children to recognise their body can do lots of different things and people of all shapes and sizes can enjoy dancing. Notably, in line with social learning theory ( [54]) the child actors are older than the target audience to serve as positive role models for younger audiences to look up to.

#### Micro-intervention 2: ‘My Bbody is Amazing’–music video

The series ‘Blippi’s Wonderful Talent Show’ will be complemented by a collection of music videos, all of which will be hosted on YouTube and additional music streaming platforms. The choice to evaluate one music video was primarily made due to funding constraints and considerations surrounding the availability of the final produced versions of the videos within the confines of the project timeline.

‘My Body is Amazing’ is a 3-minute up-beat music video. The song teaches children about the various functions of different body parts, particularly the senses, with repetitive and ‘sticky’ lyrics (e.g., ‘*boom boom*,* what’s that sound? We can work it out! My ears can hear the music playing all around)*. The key aim is for children to recognise their body has value, beyond its appearance. It stars Blippi, Meekah and a girl actor aged nine.

A detailed outline of the intervention development process is presented in Table 2.

**Table 2:** Intervention development process

May 2023 - July 2023.	Literature Review
	• Body image research in early childhood remains a relatively underexplored area. Longitudinal studies highlight that from age 3 years children express negative attitudes towards others [7], at age 4–5 years display relatively positive body image but by age 6 a large proportion (up to 70%) report signs of body dissatisfaction [9]. • Three successful strategies (a storybook, a theatre production and short animations) to foster positive body image among young children were identified [45–47]. • Media micro-interventions have demonstrated their efficacy in improving body image among children [46, 48] and are suited to young children’s limited attention span. They are also cost-effective. • A comprehensive overview of body image and related measures among young children were collated, which indicated limited availability of appropriate measurement tools. • Based on our literature review, bolstering positive body image and reducing weight bias were identified as the focus of our interventions.
July-September 2023.	**Public Involvement (PI)**
	• PI experts (children, parents, teachers and a body image expert specialising in young children) were recruited with the aim of informing the research protocol. **Parents and Teachers**: • In relation to media viewing habits, parent interviews indicated young children typically watched TV and online videos for 15–20 min (i.e., short episodes) and enjoyed interactive programmes focused on learning. Parents indicated some interest in co-watching children’s media with their child. • With regard to how children talk about bodies, teachers and parents said children talk about bodily functions or its physicality (e.g., they can run fast). Some were able to provide examples of instances where children have made negative comments about how others look (specifically in relation to skin shade, or weight). Children talking negatively about their own bodies and how they look was less frequent. • Parents and teachers also provided feedback on aspects of the proposed research design to ensure processes were age appropriate, acceptable, and practical. This included giving the research team direction on how to question children on the topic of body confidence, under what conditions we should show the intervention, and how long we can expect to keep young children engaged. **Children**: • Potential outcome measures were tested among the children’s PI group through three rounds. Based on the teams’ (NC, HS and KG) observations during these sessions, refinements to the wording, number of items and response scales were made. A final shortlist of questions was determined. • Final versions of the materials and outcome measures were reviewed by two teachers. • Final adjustments included adding a sense-check question into the interview schedules (e.g., ‘Oh you’ve stood there, so you think X’) and final tweaks to the instructions (e.g., changing ‘does that make sense to you?’ to ‘Does that sound OK to you?’).
July-September 2023.	**Intervention Format**
	• Moonbug Entertainment identified 4–6-year-olds as key consumers of Blippi content. • Initially, Moonbug Entertainment presented the idea of developing a 60-minute ‘movie’. However, after reviewing viewership data from Moonbug Entertainment’s team and initial insights from PI work, short episodes and music videos were chosen as appropriate intervention formats for testing.
July – August 2023.	**Intervention Messaging**
	• Two key themes under the umbrella of positive body image [1, 51] were established: (1) functionality appreciation – e.g., focusing on what the body can do, as opposed to its appearance [52, 53] and (2) acceptance of diverse appearances – e.g., recognising that people come in various shapes and sizes, and everyone is of equal worth. • It was decided that positive body image psycho-educational messaging would be delivered both explicitly (i.e., through the lines in the 15-minute episode script and music lyrics) and implicitly (i.e., via casting and additional special effects graphics). This is an effective learning strategy for young children [40].
July – August 2023.	**Creative Concepts**
	• Moonbug Entertainment developed the initial concept of a ‘talent show’ whereby children meet various experts (e.g., magician, inventor, and dancer) and together they explore and discover how wonderful their bodies are in a fun and creative way. • The music videos are also based on the talent show; to supplement the 15-minute episode and each one includes positive body image messaging.
July 2023 – November 2023.	**15-minute Episode Script and Song Lyric Development**
	• The development of the scripts and music lyrics was an iterative process between all stakeholders. • For the episodes, an initial storyboard outline and narrative was developed by Moonbug Entertainment and reviewed by the research team (NC, KG, HS, HW). Once all stakeholders were aligned, a full script was written with the aim to promote positive body image in a fun and interactive way, incorporating the two themes identified above. • An example edit to the script: changing the lines *‘there’s a fun dance move that might be better suited to your body…’* to *‘there’s a fun dance move that you might enjoy more [or] that suits your style more’* to avoid the implication that certain body types are better suited to certain dance moves. • In tandem, outlines for 8 music videos were drafted. Each one represented a key theme within the umbrella of positive body image and represented a different style of music (e.g., pop, rock). An additional 9th song was developed for the purpose of the storyline of the Talent Show but did not include key messages on body image.
August 2023 – September 2023.	**Casting**
	• In line with principles of Positive Body Image Theory [1, 53] and to disrupt gender and appearance stereotypes typically seen in children’s media [16], it was essential that actors were diverse in body size and shape, ethnicity, and gender. • To reinforce the key messaging in the script, the cast (both adults and children) modelled body acceptance and appreciation by portraying their bodies positively, talking positively about their bodies and appreciating what it can do for them [38]. • Once casting briefs were finalised and sent out, auditions were held, all stakeholders reviewed the casting shortlist, and selections were made for six additional characters ( in addition to the shows' two main characters, Blippi and Meekah). • Actors were deliberately cast to show a range of body sizes, including those that are not typically represented in mainstream media (e.g., the KID DANCER, will be a boy in a larger body, disrupting gender and body-type stereotypes seen in children’s media and INVENTOR EXPERT will be a Black woman in a larger body, disrupting gender stereotypes).
September 2023.	**Pre-Production**
	• Moonbug Entertainment hosted a virtual table read of the script (via video conferencing) for the four episodes . The actors who play Blippi and Meekah read the script aloud alongside members of Moonbug Entertainment who filled in other roles. Collaborators (from Dove and authors NC and HW) watched and had opportunity to make notes and recommend changes after the event • The primary purpose of the table read was to ascertain episode length. The script was too long thus it was streamlined slightly.
October 2023 – November 2023.	**Production**
	• The episodes and the first five music videos were shot in LA, USA over ten days. Shooting included four locations, one for each scene. • The last author and research lead (NC) attended each day and consulted on the delivery of the script to ensure key messages were delivered and emphasised. • A second two-day shoot was held for the remaining four music videos.
October -November 2023.	**Post-Production**
	• Children are visual learners [40] therefore it was important to supplement the content of the script and lyrics with additional visuals. These included special effect overlay graphics (e.g., when talking about noses, different shapes and sizes of noses will pop up on the screen) and colourful word pop ups (e.g., ‘body’). • Close-to-final versions of each media were delivered towards the end of November 2023, for the pilot study to take place. • Final versions will be delivered at the end of December 2023/beginning of 2024.

### Control content

#### Control 1: ‘BlippiGgoes to the Dentist’ – 15-minute episode

The 15-minute control group will watch a Blippi episode containing no body image or appearance-related messaging. In this episode, Blippi goes to the dentist for a check-up and learns the importance of oral hygiene. This episode was chosen for its comparable length (e.g., 14 min), similar format with the intervention episode, and its alignment with the disclosed study theme in the parent information sheet: evaluating the impact of children’s media on children’s well-being self-esteem.

#### Control 2: ‘Blippi Brushes his Teeth’ – music video

The music control group will watch a 2 min 29-second Blippi music video containing no body image or appearance-related messaging. This music video is up-beat and features Blippi brushing his teeth. This music video was chosen for similar reasons as above; it featured the same Blippi actor as our intervention music video, was comparable in length and format, and was thematically aligned to the topic of children’s media, well-being, and self-esteem.

### Public involvement

Due to the limited body image research available among 4–6-year-olds, public involvement was imperative to the research design of this project. The public involvement group consisted of children aged 4–6 years old (*n* = 10; 6 girls, 4 boys), parents of children aged 4–6-years-old (*n* = 5, all mothers), early years schoolteachers (*n* = 2), and we also consulted with an expert in children’s body image. The group informed the research materials outcome measures and the study protocol. For more detail on how and when we worked with the public involvement group see Table 2.

### Outcome measures

All child measures have been purpose built or adapted from existing measures validated for use among older children as there are none appropriate for the lower end of our target age group (i.e., 4 years old). As recommended by Clark ( [55]), they will be administered in a play-style interview with a trained moderator in a 1:1 format (e.g., one moderator per one child) and answers recorded by the moderator on a reporting form. To make the interview child-friendly and to aide children’s honesty when answering, the measures will be framed as games. Play-based approaches are recommended for this age and by framing the measures as games and an opportunity to play, we hope to reduce social desirability bias [56].

### Child measures

#### Body appreciation

Body appreciation, will be assessed using two items; ‘*do you love your body?*’ and *‘do you think your body is amazing?* Both items will be asked using a two-step approach. Taking the two questions in turn, participants will first be asked to respond: ‘*yes*’ or ‘*no’*. To make this more playful, participants will be asked to respond by standing on rectangles labelled ‘*yes*’ or ‘*no*’, which the moderator will point out. To provide greater variation in children’s responses, if the child responds ‘*yes’*, they will be asked a follow-up question to indicate *how much* they love their body and *how* amazing they think their body is, with three response options: ‘*a little bit’*, ‘*a medium bit*’, or ‘*a lot*’. Participants will be asked to respond by standing on one of three circles placed in front of them to indicate how much: a small circle (approx. 20 cm in diameter) will represent ‘*a little bit’*, a medium circle (c.30 cm in diameter) will represent ‘*a medium bit*’, and a large circle (c.40 cm in diameter) will represent ‘*a lot’*. The circles will be one colour, be presented on the floor in front of them from smallest (*left*) to largest (*right*) for them to stand on. To prevent potential influence from their previous responses, participants will stand on a ‘*start*’ rectangle before answering each question.

**Fig. 2:**
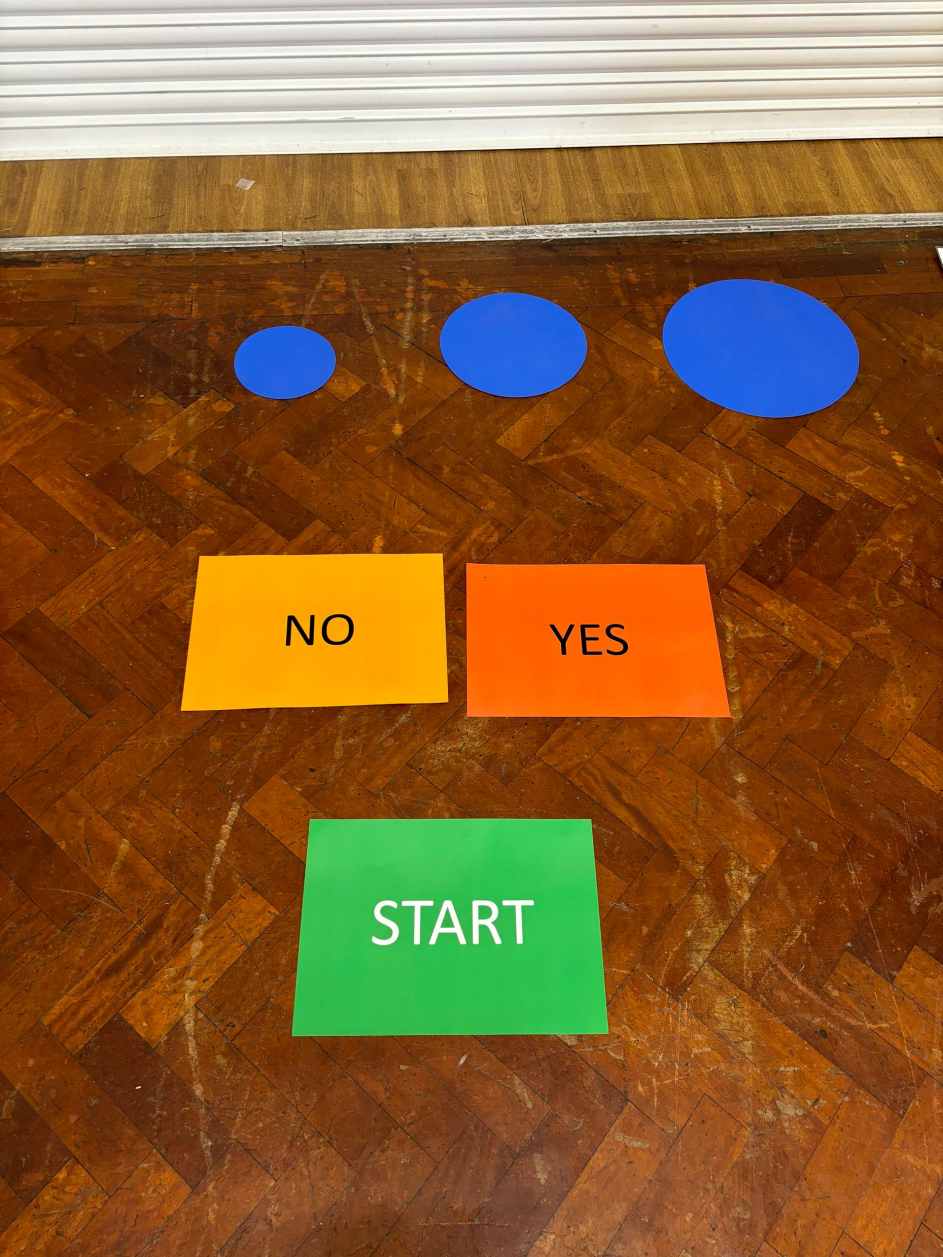
Body appreciation measure - ‘circles'

Possible scores range from 1 (*no*) to 4 (*a lot*). A mean of the two items will be calculated with higher scores indicating higher body appreciation. See Fig. 2 for a visual representation of the response scale for this measure.

The two items (listed above) were chosen for both practical and comprehension reasons considering the existing literature and our public involvement insights. First, the research team (NC, HS, KG, HW) collated a list of eight items (see Supplementary File 1) from the two most widely used body image measures for children; the Body Appreciation Scale-2 (BAS-2 C; [57]) and Body Esteem Scale for Children (BES-C; [58]). These items were selected as the most age appropriate for 4–6-year-olds as well as mapping closely onto our intervention’s key messages. Children aged 4–6 years can effectively respond to circles of graduate sizes to indicate their response ( [59]) and Alan and Kabasakal [60] recommend nothing more than a 3-point response scale for this age group.

We first adapted the format of the items from statements (e.g., *I like how I look*) to questions (e.g., *do you like how you look?*) for age-appropriateness. Then, we tested candidate items with our child, parent and teacher public involvement groups. We found children struggled to differentiate between some of the items (e.g., ‘*do you like how you look?’* and ‘*do you like your body’*?) or had poor comprehension of abstract concepts (e.g.,* ‘do you think you have a good body?*’). Other items such as ‘*do you like what you look like in photos?’* and ‘*do you like what you see when you look in the mirror?*’ were conceptually too challenging, with some children requesting a concrete example of which photograph, or which mirror and when. This aligns with previous research among this age group [59].

Finally, it was evident the attention span of children this age was short, owing to the increasing despondent responses from the children the longer the activity took (e.g., child would lose interest, hop around, stare at things on the wall, play with the materials, or tell the moderator they were bored). To circumvent this, the decision was made to use two items to assess body appreciation. The item ‘*do you love your body?*’ was selected for being the most readily understood, broad in its messaging (i.e., not directed toward appearance or functionality), and providing the most variability in responses given by the children in our public involvement work. The second item, ‘*how amazing do you think your body is?* was chosen because it directly maps onto the intervention’s key messages. Finally, children responded well to stepping on the small, medium, or large circles to indicate their answer.

Four filler items will also be asked, dispersed amongst the body appreciation items. These will be different at each time-point. Examples include: *‘do you love broccoli?’*, ‘*do you love brushing your teeth?’* and *‘do you love playing outside?’.* The filler items will act as an icebreaker to the activity, and a mechanism to reduce social desirability [61]. Each moderator conducting the interview will praise the child for their participation and engagement, focusing on their involvement and effort rather than a specific response, to avoid influencing their answers.

This measure will be called ‘Circles’ to the participating children.

#### Functionality appreciation

An adapted version of a measure used by Swami et al. [47] and Dohnt and Tiggemann [45] among a similar age group will be used to measure functionality appreciation. The measure is open-ended and asks children to; *‘tell me all the amazing things you can do with your body’*. In line with Swami et al. [47] and Dohnt and Tiggemann [45] children will be allowed to respond with as many answers as they wish. The moderator will ask the question then will turn over a 60-second sand timer. Children can respond with as many answers as possible before the sand runs out. If a child is still talking when the sand timer had finished, they are allowed to continue until they coame to a natural stop. Responses will be recorded verbatim.

This measure has been refined through several rounds of public involvement with children, teachers, and parents. The wording used by Swami et al. [47] and Dohnt and Tiggemann [45] differed slightly as their questions directly related to the theme of the intervention being tested. For example, Swami et al. [47], who evaluated a theatre production of Cinderella *(‘Cinderella: the AWESOME truth’*) which encouraged children to recognise their own awesome and unique qualities, asked children to list *‘what is unique about you?*’. Dohnt and Tiggemann [45] who evaluated a positive body image storybook focusing on special talents asked children to list ‘*what special talents do you have?*’. Our interventions encourage children to recognise the amazing things their body can do. Therefore, we tested the following item with our public involvement group for comprehension; ‘*tell me as many things as possible that your body can do. You can say things that happen inside your body or things different body parts can do – you can say anything your body does for you!’.* These instructions were evidently too lengthy. Consequently, a simplification of the wording was implemented with the addition of the term ‘amazing’ to qualify responses. An absolute count score will be calculated, with a higher number of responses indicating higher functionality appreciation. In addition, content analysis will be conducted, exploring the content of children’s answers to strengthen the interpretation of our findings.

This measure will be called ‘Time’ to the participating children.

#### Weight bias

**Fig. 3:**
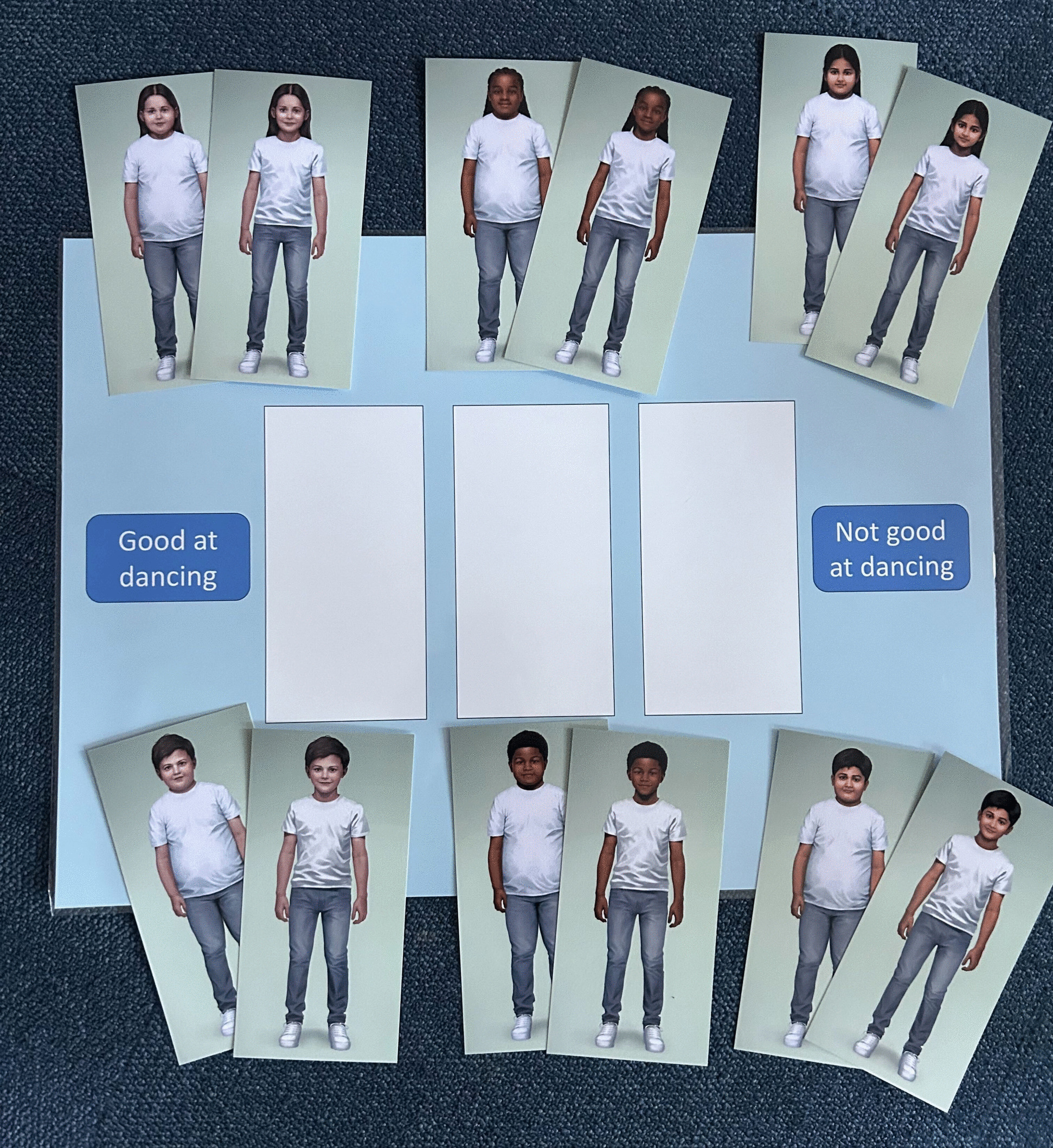
Weight bias measure - 'pictures'

To measure weight bias in children aged 4–6 years, existing measures developed by Parnell et al. [62] and Damiano and colleagues [63] for use among young children were adapted. Children will be asked to rate both a smaller and a larger child (matched on ethnicity and gender; see Fig. 3) on a 3-point scale containing two bipolar adjectives or phrases. There will be five pairs in total, presented in the same order each time; very clever/not clever at all, cute/ugly, has no friends/has lots of friends, active/not very active and good at dancing/not good at dancing.

The child images were created by illustrator, Tom Waterhouse, for the purpose of this study. Tom previously worked with Parnell et al. [62] and Matheson et al. [46] to develop a set of child images to assess appearance stigma among primary school children and 7–14-year-olds, respectively. For the present study, there will be three sets of made-up images in total that broadly represent three ethnic groups: Black girl and boy, South Asian girl and boy, and White girl and boy. These groups were chosen as they represent the three largest ethnic groups in the UK [64]. Each set of images includes a smaller and a larger child dressed in the same clothes (e.g., plain t-shirt and jeans with white trainers). They are stood up straight, hands by their sides with a slight smile on their face.

The response scale for this measure was reduced from a 7-point [63] to a 3-point ordinal scale, ranging from 1 = *child rates the image positively*, to 3 = *child rates the image negatively.* The middle option, a score of 2, is given when a child rates the child image *as ‘somewhere in the middle’*, indicating a *neutral* response. A total score will be calculated by the mean difference between smaller and larger child, with higher scores reflecting higher weight bias.

This measure will be called ‘Pictures’ to the participating children.

### Intervention acceptability

We will also examine child and parent intervention acceptability across all conditions at post-intervention (T2).

Children will be asked three acceptability questions. Children will respond using the same rectangles and circles as per our body appreciation measure to the question: ‘*did you like the [episode/music video] you just watched?*’ whereby children stand on a ‘start’ rectangle and the moderator will ask them to respond, ‘*yes’* or ‘*no’*. If the child responds yes, they will be asked ‘how much’ using three response options: ‘*a little bit’* (smallest circle*)*, ‘*a medium bit*’ (medium circle), or ‘*a lot*’ (biggest circle). They will be encouraged to expand on their answer giving details of what they did/did not like.

To check children’s familiarity with the media content, we will also ask children to answer; ‘*yes’* or ‘*no’* to; *‘have you seen this [episode/music video] before?’*. Finally, children will be asked an open-ended question to explore their learnings from the content they watched; ‘*did you learn anything while watching the [episode/music video]?’.*

Parents will complete a paper-based questionnaire containing questions related to the content they watched. Parents will be asked; ‘*how much did you like the episode/music video?’* with responses on a 5-point scale ranging from 1 *= did not like it at all* to 5 = *liked it very much.* They will also respond to; *‘Do you think this message is important for your child to learn at their age?’* and *‘Would you recommend this episode/music video to other parents of children of a similar age?* on a 3-point scale *yes*,* no* or *unsure.* Several open-ended questions will allow parents to expand on their answers (e.g., things they *did/did not* like).

#### Repeat watching

In between post-intervention (T2) and follow-up (T3), parents will be given access to their assigned media content via a secure link and encouraged to rewatch it with their child before they return for their next session in approximately seven-ten days’ time. Parent’s will self-report how many times they re-watched their content in their T3 follow-up survey.

#### Child interview moderator fidelity

All children’s interviews (T1, T2 and T3) will be audio recorded for the purpose of assessing moderator’s adherence to and competency in delivering the child interviews across each timepoint. The adherence checklist will align directly with the child interview protocol which all moderators have received training on. Moderators will also be rated on seven dimensions of competency (e.g., organisation, communication and expression, friendliness).

Overall, the pilot study will involve interviews with 80 children across three timepoints (total of 240 interviews) and the larger scale RCT will involve interviews with 440 children across three timepoints (total of 1320 interviews). 20% of interviews from the pilot (i.e., 48 interviews) and 10% of interviews from the main RCT (i.e., 120 interviews) will be selected. A proportion (40% and 20% for the main trial and pilot, respectively) will be double checked to calculate inter-rater reliability. Adherence will be determined by how closely the moderator follows the protocol (expressed as a percentage), and competency will be calculated by a mean score for each competency dimension.

#### Eligibility criteria

The micro-interventions are intentionally designed to be universally applicable, therefore children will not be screened for the presence or absence of positive body image. Eligible participants are; English-speaking children who are aged 4, 5 or 6 years old in Reception Class or Year 1 at primary school, with a respective parent/guardian located in Birmingham or Greater London. Participants will be excluded if they are a sibling of a child already recruited into the study (i.e., only one child per household can take part), and children who have complex special educational needs that would hinder their ability to enjoy partaking in the intervention(s) or the research process.

#### Recruitment

Recruitment will be led by a research agency (We Are Family) who have 25 + years of experience conducting research with children and their families. In the period of two to eight weeks prior to data collection, study information will be sent out to potential parents via We Are Family, which included details of participation and the purpose of the research: to evaluate the impact of children’s media on children’s wellbeing and self-esteem. Parents were not told the media being tested was Blippi. Parents of eligible children will be recruited based on three quotas: (i) an equal number of girls and boys, (ii) an equal number of children in Reception and Year 1, and (iii) at least 20% of children belonging to ethnic minority groups. Interested parents will be asked to complete a brief screener questionnaire including demographic information (e.g., age, ethnicity, employment status, socio-economic status) and eligibility criteria. If eligibility criteria are met, they will be asked to provide digital informed consent for them and their child to take part in the study. Once consent is gained, they will progress into the study. Those that do not consent will be thanked for their interest and will not proceed. Participants will then be randomised into one of four conditions by We Are Family using a spreadsheet detailing the block allocations. Following allocation, We Are Family will give parents the details of the location and time for the data collection and media screening sessions.

### Procedure

We Are Family will be responsible for contact with parents and organising study logistics (e.g., booking venues). The data collection and media screening sessions will be conducted face-to-face, in person with a team of moderators trained to deliver the research protocols (by KG and NC). There will be approximately 12 children with one of their respective parents per session. At T1, participants will arrive and be welcomed to the venue. The venue includes an initial waiting area and a main space where the child-interviews will take place. The main space will comprise of 12 booths around the edge, each with a table and two chairs, including a chair placed outside the booth for each parent to sit on. Once welcomed, participants will engage in five minutes of group play, facilitated by a moderator, to allow children to relax and become familiar with the environment. Next, participants will engage in a brief play activity with their allocated moderator to act as an ice breaker to the interview schedule. A moderator will complete the pre-interview (T1) with their assigned child (i.e., one moderator to one child) while their parent is encouraged to sit outside the booth. The pre-interview will commence with the child providing verbal assent (they will be told what to expect of the session and asked if they are happy to take part) and is expected to last up to 15 min.

As soon as the child has completed their interview, they will be given a tablet and two sets of headphones (one for the child and one for the parent) and asked to watch their assigned media together however they choose (e.g., sat on the floor, standing, on a chair). The moderator will remain close to the booth to provide any assistance if needed but will primarily be noting down any observations (e.g., engagement levels). Those in the music video conditions will be instructed to watch a 10-minute neutral video (i.e., Numberblocks) on a tablet, before they view their music video. This is to ensure each condition moves on to the post-interview at roughly the same time.

Immediately following the screening of their allocated media content, children will complete the post-interview (T2), with a moderator. While this is happening, parents will be asked to complete a paper-and-pen questionnaire. Once finished, participants will be thanked for their time and children will receive a small gift (e.g., stickers). Each parent will receive a secure link to their assigned media and will be encouraged to watch it again with their child at home over the next seven to ten days. Snacks and water will be available for children to have before, during, or after the session.

**Fig. 4:**
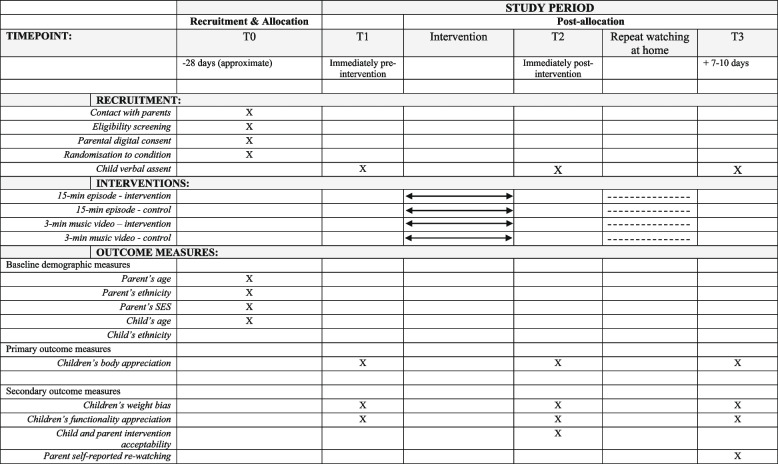
Schedule of recruitment, interventions, and outcome measures (SPIRIT)

Approximately seven to ten days later, children will attend a follow-up session (T3) accompanied a parent. Children will engage in a follow-up interview with a moderator (i.e., the same format as T1 and T2). Following the completion of this third interview, parents will be provided with a debrief form which will include the explicit study aims, the condition they were allocated in, and details of mental health resources available for parents and children. Finally, to compensate participants for their time, We Are Family will organise thank you incentives. Parents will receive £130 in the form of cash payment, voucher, or donation to a charity of participant’s choice, and children will receive another small gift (e.g., stickers) and a certificate. See the schedule of enrolment, interventions and assessments in Fig. 4. The SPIRIT checklist is presented in Supplementary File 2.

### Statistical power and sample size

A proposed sample of 440 children aged 4–6 years and a corresponding parent will be recruited for this study. The study has been powered for immediate post intervention effects to be assessed using analysis of covariance with the commensurate baseline measure as the covariate. Caution has been exercised for assumed for effect size due to a universal non-clinical population undergoing a micro-intervention. This is offset by an assumed medium to large pre- post- correlation due to the short time interval between measures and the high degree of internal validity at data collection. For a two-sided test (alpha = 0.05), a sample size of *n* = 103 per arm will have 90% power for an assumed standardised effect of Cohen’s delta = 0.3, with pre-post-correlation of 0.75. A sample size of *n* = 92 per arm will have 80% power for a standardised effect of Cohen’s delta = 0.25 with pre- post- correlation of 0.8. The aim is to sample for *n* = 105 per arm (90% power, *r* = 0.75, *d* = 0.3). To achieve sample size, the target recruitment will be inflated to *n* = 110 per arm to compensate for any missing data.

### Statistical analyses

At trial completion, the data will be entered into SPSS, cleaned, screened and the T2 acceptability data separated out for concealment purposes. The concealed dataset will be delivered to the statisticians to conduct analyses.

The primary analysis will be on an Intention-to-Treat (ITT) basis inclusive of all randomised participants who provide some outcome data either immediate post-intervention (T2) or at follow-up (T3). Conclusions will be drawn from the ITT analysis set. In addition, a supplementary per-protocol analysis set, excluding any protocol deviations, will be undertaken. Prior to inferential analysis there will be a validity and verification assessment for data veracity. The primary outcome measure is body appreciation with endpoint being body appreciation immediate post intervention (T2). The primary analysis will be an analysis of covariance comprising randomised arm and body appreciation at T1 as a covariate. This model will include a baseline by covariate interaction term if necessary for model validity. Underpinning assumptions will be assessed for model validity and remedial action taken if required (e.g., robust homoscedasticity corrected analyses or bootstrap approaches as deemed necessary to ensure valid statistical conclusions). If the data is highly discrete to the extent of potentially compromising statistical conclusions, then the linear link function will be replaced by a more appropriate link function (e.g., ordinal logistic regression or negative binomial regression). If the baseline by covariate interaction term is deemed important then a follow-up Johnson-Neyman analysis of covariance will be used. These analysis of covariance models will compare (i) music intervention and music control using the data from the two appropriate arms, and (ii) 15-minute episode intervention and 15-minute episode control using the data from the two appropriate arms. Effect sizes, 95% Confidence Intervals and standardised effects will be reported as appropriate.

The above analyses will be extended to assess any moderating effects by gender, and any moderating effects by year group.

Secondary exploratory analysis will compare the 15-minute intervention and music intervention using the data from the two appropriate arms using the same methodological process.

The above analysis plan for body appreciation will be repeated for the secondary outcome measures of functionality appreciation and weight bias. Secondary research questions at T3 will be examined using the same analysis of covariance methods controlling for baseline (T1).

The aim is to have ITT data on *N* = 420 participants. This will be achieved through a target recruitment of *N* = 440 cognisant that some potential participants might be unable to partake in the research due to external mitigating reasons. If the size of the ITT analysis set is N > = 380 then the data will be analysed as given. If the size of the ITT analysis set due to missing data is between 260 and 380 then a full missing values analysis will be undertaken, and appropriate sensitivity analyses performed. If the size of the ITT analysis set due to missing data is less than 260 then results will be given descriptively.

### Additional analyses

#### Acceptability analysis

Children’s acceptability will be calculated by the percentage (%) of ‘*yes’* and ‘*no’* responses and the 3-point scale will be calculated as a mean. In line with the children, parent’s acceptability will be calculated by the percentage (%) of ‘*yes’*,* ‘no’ and ‘unsure’* responses. For the 5-point scales, a mean score will be calculated.

#### Qualitative analysis

Content analysis will be used for all open-ended questions (e.g., functionality appreciation and child and parent acceptability).

For functionality appreciation, two checkers (KG and HS) will independently assess children’s responses at each time point (T1, T2 and T3), determining which are valid based on the theoretical framework and conceptualisation of functionality appreciation ([52]). A valid response should fit into one of the six defined domains: (1) bodily senses and sensations (e.g., see, hear, smell), (2) creative endeavours (e.g., drawing), (3) internal processes (e.g., breathing), (4) physical capacities (e.g., playing games, sports), (5) communicating with others (e.g., hugging) and (6) self-care (e.g., brushing teeth), through importantly through the worldview of a child. Duplicate responses (e.g., walk and walking) will be collapsed and counted as one. Invalid responses include simply naming body parts (e.g., legs, arms) or non-sensical responses that do not fit the categories listed above. We will calculate a two-way mixed-effects, single-rate, absolute agreement ICC [65] to indicate if scoring is consistent between raters. A score of at least 0.75 indicates good reliability [65]. A total score for functionality appreciation will be the sum of valid responses, with higher scores indicating higher functionality appreciation.

### Ethical approval and trial registration

This study has received Research Ethical Approval (CHSS.23.09.021) from the College of Health, Science and Society, at the University of the West of England, Bristol. The trial is also registered with Clinical Trial.gov reference number: NCT06146647.

### Data management, data availability and sharing

Each participant will be given a unique participant ID for the purpose of matching responses over time. Anonymous data from participants (children and parents) from each timepoint (T1, T2 and T3) will be collected via paper and pen and entered into SPSS by members of the research team (HS, KG). The data file will be stored on a university approved cloud storage (e.g., OneDrive) and hard copies stored in a locked cabinet in line with the university’s policy. Hard copies will be screened for personal information and anonymised if required. The data file will be screened for data quality including completeness. Digital consent data and personal identifiers (e.g., participant contact details) will be collected by the research agency and kept securely until the end of the project; this information will not be shared with the authors. Audio recordings will be securely uploaded, cross-referenced and deleted once fidelity checks and inter-rater reliability assessments have been completed and resulting paper has been published.

An independent data monitoring committee will not be used as this project is deemed low risk and data collection will take place in a short period (a few weeks). Further, research team members who are not be concealed from condition allocation (i.e., not the statisticians) will review the data as it accumulates to assess for risk. The pilot also allows for a safety assessment of the trial.

The funder and the research agency will not have access to the raw data and all parties have a data processing agreement in place. Anonymised data will be made available upon reasonable requests for non-commercial use. Requests should be made to the project lead and corresponding author (NC).

### Dissemination

Providing there is no evidence of harm, the two interventions will be uploaded to Blippi’s YouTube channel, which has over 22 million subscribers on YouTube (as of December 2024). Therefore, there is huge scope for reaching children around the world in an organic way.

The findings from this study will be disseminated in peer reviewed publications, delivered as presentations at relevant academic conferences, and shared among the social media outlets, podcast episodes and websites.

## Discussion

Micro-interventions offer an innovative approach for targeting young children with positive body image content and may help to mitigate the onset of body image concerns in later childhood. They can be easily embedded into children’s media environments (especially online platforms) thereby disrupting the ubiquitous problematic content that currently permeates these spaces. Directing positive body image content towards children as young as four is potentially a cost-effective preventative strategy for targeting mental health concerns at scale. This study protocol reports on the development and planned randomised controlled trial of two positive body image media micro-interventions among young children aged 4–6 years old. To our knowledge, these are the first interventions designed to promote positive body image among children as young as four, with no published work having explored the medium of music video among this group.

The proposed study has numerous strengths, addressing previous gaps within the literature. Firstly, the proposed randomised controlled trial (RCT) design has been rigorously developed, fully powered, and with the inclusion of active control groups; the gold standard for intervention evaluations [66]. We also include a one-week follow-up period to assess the short-term effectiveness of the micro-interventions and explore any dose response or delayed effects, building upon the preliminary micro-intervention work which has been conducted in field of body image. Secondly, the utilisation of several public involvement groups within the development of the interventions but also the research process only strengthens the rigour of our study. Thirdly, our sample will be gender inclusive, extending the groundwork laid by Dohnt and Tiggemann [45] who were the first to assess a media based positive body image intervention (i.e., storybook) for young children but exclusively among girls. Finally, our recruitment pool will span Birmingham and London, the UK’s two largest cities, both of which diverse in terms of ethnicity and socio-economic status.

Despite several strengths, there are some limitations, mainly related to the challenges of working with very young children. First, scant body image measures were available for administration among 4–6-year-olds. As such, the measures described in this paper were purpose built or were adapted from the few age-appropriate measures available. Although the measures were carefully developed with body image experts, informed by current evidence and several rounds of public involvement feedback, further research beyond the scope of this study will be required to conduct a rigorous validation to examine their psychometric properties. We believe this is an important next step in being able to advance the understanding of young children’s body image.

Similarly, the quality of the self-report data we obtain from this age group may vary. Due to children’s developmental capacity around ages 4 to 6 years, uncertainties persist regarding the amount of unusable data that may be collected (e.g., non-sensical responses, child refusal to engage or interact, or simply a lack of comprehension of the questions). From the beginning of the project, our measures and processes were thoroughly informed by public involvement work to mitigate the likelihood of unusable data. Nevertheless, an awareness that some participant responses may be unusable has been factored into our power calculations. It is hoped that by conducting this work and being transparent in our research endeavours, we can shed light on these issues for other researchers to learn from.

Designing and evaluating interventions through a collaborative process between academics, creatives partners and industry as described in this protocol paper is hugely beneficial for many reasons. All stakeholders bring different skills and expertise, together ensuring interventions are developed to be as acceptable and enjoyable to the target group as possible, while also grounded in a scientific evidence-base. Academic partners bring the scientific knowledge including current evidence on attitudes and behaviour change, as well as rigorous research methods. The creative partners bring the academic messaging to life through storytelling, animation, and humour. Finally, industry partners can maximise intervention reach. If proven to be effective, these stand-alone media micro-interventions have potential to reach hundreds of thousands of young children in many countries across the world.

## Supplementary Information

Supplementary Material 1.

Supplementary Material 2.

## Data Availability

No datasets were generated or analysed during the current study.

## Abbreviations

UK: United Kingdom
RCT: Randomised Controlled Trial
T1, T2, T3: Time One, Time Two, Time Three
SES: Socio-economic Status
TV: Television

## Footnotes

1 The Tripartite Model of Influence [13] is a psychological framework to explain how individuals develop and internalise appearance ideals. The model suggests peers, family and the media contribute to the onset and development of body dissatisfaction through two mechanisms: appearance ideal internalisation and upwards appearance social comparisons.
