# Supplementary material for: Evaluating the effectiveness and acceptability of two positive body image media micro-interventions among children aged 4–6 years old – a study protocol
Source: BMC Public Health. 2024 Dec 19;24:3539. doi: 10.1186/s12889-024-20869-z (PMC11660667; doi:10.1186/s12889-024-20869-z)
Supplement: Supplementary file 1 — Supplementary Material 1. [file 12889_2024_20869_MOESM1_ESM.docx]

**Supplementary File 1.** Collated Items from the BAS-2C [57] and BES-C [58] for public involvement testing.

1. Do you like your body?
2. Do you feel love for your body?
3. Do you feel happy about the way you look?
4. Do you like how you look?
5. Do you feel good about your body?
6. Do you think you have a good body?
7. Do you like what you look like in photos?
8. Do you like what you see when you look in the mirror?
